# Is there a link between depression, neurochemical asymmetry and cardiovascular function?

**DOI:** 10.3934/Neuroscience.2020022

**Published:** 2020-09-28

**Authors:** AB Segarra, I Prieto, M Martínez-Cañamero, Manuel Ramírez-Sánchez

**Affiliations:** Department of Health Sciences, University of Jaén, Jaén, Spain

**Keywords:** brain asymmetry, brain-heart connection, brain-gut connection, hypertension, neurovisceral integration, neuropeptidases

## Abstract

Although at present depression is one of the most disabling disorders in our social environment, the understanding of its pathogenesis and the resources for its treatment are still unsatisfactory. The importance of brain asymmetry in the pathogenesis of disorders in brain function, including mood disorders such as depression, is a highly unexplored, sometimes underrated, and even ignored topic. It is important to note that the basal and pathological functional lateralization must have an underlying neurochemical substrate. It is also necessary to indicate that the brain asymmetry extends to a neurovisceral integration whose behavior may also be lateralized. One of the most studied axis from the functional point of view is the brain-heart connection, in whose operation there are observations that suggest an asymmetric behavior in basal conditions that is modified by central and peripheral changes, as well as by pharmacological treatments. There are evidences that connect cardiovascular function, neurochemical asymmetries, and depression. A deep understanding of the bilateral behavior of the brain following pathophysiological changes in blood pressure as well as pharmacologically induced, can provide us with therapeutic suggestions for the treatment of depression. In this article, we analyze remarkable results of some representative selected contributions, with which we discuss our proposal on the relationship between hypertension, depression and neurochemical asymmetry.

## 1. Depression and brain asymmetry

Depression represents a major mental health problem that can involve neurochemical, neuroendocrine, anatomical, and cognitive changes [1]. These include abnormalities in the cerebral monoaminergic content, various neuroendocrine manifestations such as alterations in the hypothalamic-pituitary-adrenal axis, anatomic cortical, limbic, striatal and white matter alterations, and cognitive disorders that involve processes of perception, attention, memory and reward [1]. However, it also seems clear that many other factors may be involved in its pathogenesis. In general, there is an underlying neurovisceral alteration that reaches multiple systems such as the brain-heart connection or even the gut-brain axis [1]–[4]. The multivisceral response to stress seems to have a central role in the development of depression [5]. Chronic stress alters the neural interactions between cortico-limbic areas, especially the prefrontal cortex and the hippocampus, directly involved in the development of depression [5]. Acute stress also modifies the interactions between cortico-limbic areas [6] and is also considered to participate in the development of depression [7],[8].

Currently, antidepressant treatment, mainly with tricyclic antidepressants, is based on the inhibition of the reuptake of monoamines (serotonin, norepinephrine) at the synaptic level, therefore increasing their availability [5]. However, the efficacy of a treatment exclusively based on the use of these drugs is not entirely satisfactory and other ways of therapeutic approaching are being investigated. Thus, for example, various studies have drawn attention to the possible importance of changes in the bilateral brain profile, especially of cortico-limbic areas, in the pathogenesis of depression, as well as the role of certain types of diets.

## 2. Integrative neurovisceral asymmetry

**Figure 1.**
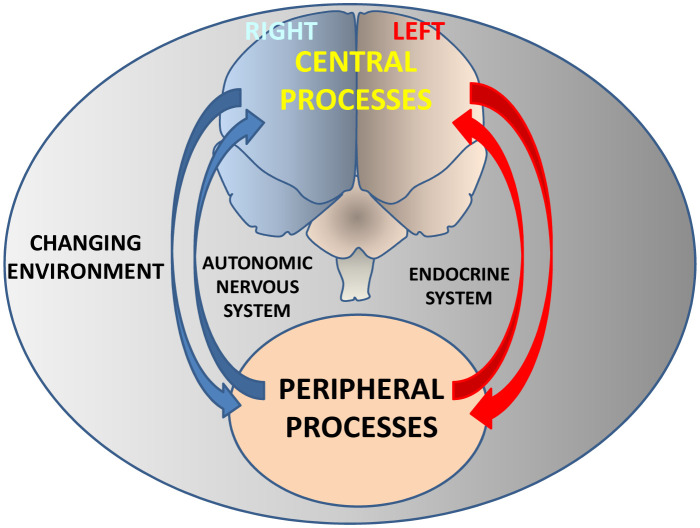
Neurovisceral integration.

To our knowledge, the first reference to the existence of a cerebral asymmetry comes from an assessment that Paul Broca made in 1861 with a certain caution's tone: “This subject, both physiological and pathological, deserves more attention than the one that most doctors have given so far, and the subject is quite delicate, the subject is quite dark and quite complicated... The disease is the result of an injury to one of the anterior lobes of the brain... Our observation places in these lobes the seat of the faculty of articulated language...” [9]. Since then, numerous investigations, mainly anatomical and functional, have been carried out, which have resulted in excellent compendia on brain asymmetry [10]–[13]. However, it seems reasonable to suppose that anatomical and functional asymmetries must have a neurochemical substrate to support them, and although this evidence appears as early as the 70s of the previous century, both in animals [14] and in humans [15], their study did not receive as much attention as the analysis of anatomical and functional lateralization [10]–[13]. All these studies have shown that brain asymmetry is present in animals, at all levels of the evolutionary scale and in humans, throughout its own evolution. It is a dynamic concept, modifiable depending on changes in endogenous and environmental factors, such as changes in light or darkness, even offering an individualized pattern depending on the interaction between central physiological or pathological processes with peripheral ones within a changing environment. This leads us to the concept of bilateral neurovisceral integration (Figure 1). Organs and tissues do not function independently in watertight compartments but interact with each other in an integrated way. One of the most studied aspects of this concept already comes from the studies of the great French physiologist Claude Bernard on the connection between the brain and the heart [16]. Less suspicious than Broca, Bernard said: “The heart and the brain are therefore in a solidarity of the most intimate reciprocal actions, which multiply and tighten even more as the organism becomes more developed and more sophisticated... The expression of our feelings is made by an exchange between the heart and the brain, the two most perfect gears of the living machine...” Furthermore, various results have shown that this neurovisceral integration can function asymmetrically, from the input of sensory information [17] to the expression of physiological functions and the generation of metabolic products [3],[18]–[20].

Hypothetical diagram showing the bilateral and bidirectional interaction between the central nervous system and peripheral tissues. The concept of asymmetric neurovisceral integration implies an asymmetric bidirectional connection between the central nervous system and peripheral organs. Through neuroendocrine mechanisms, central physiological or pathological processes will condition peripheral consequences and vice versa, peripheral processes will determine central consequences within a clear integrative vision of body function modulated by a changing environment such as, for example, variations in the conditions of light and darkness. The bilateral behavior of such central and peripheral interaction will differ depending on a multiplicity of endogenous and exogenous factors.

## 3. Depression, neurochemical asymmetry and cardiovascular function

The use of a standard treatment with antidepressants is in many cases insufficient, so new strategies from various approaches are being explored. Various evidences suggest that alterations in the bilateral basal model of brain function lead to mood disorders, including depression [21]. Similarly, it has been postulated that treatments that restore or approach the basal model of bilateral behavior would improve mood [20]. But the function is supported by neurochemical behavior and, in the case of the function that neuropeptides exert, this is reflected in the behavior exhibited by the enzymes that degrade them [22].

Framed in the concept of the brain-heart connection, the relationship between the increase in blood pressure and the appearance of mood disorders such as depression is well known, particularly in the elderly [23]–[25]. The maintenance, improvement or worsening of depression in response to different antihypertensive treatments is variable and the mechanisms are still uncertain [26]–[30]. Depression has been postulated to lead to an alteration in the bilateral brain basal function such as in the frontal cortex among other regions [31], this one especially related with cardiovascular control [19]. Improved depressive symptoms would be related to increased activity in the left hemisphere compared to patients in whom no improvement was seen [31]. It is interesting to relate this proposal with results obtained after unilateral neurotoxicologic injuries of the nigrostriatal system in rats [32]. Left injuries in normotensive animals, with the consequent left depletion of dopamine, but especially in hypertensive ones, dramatically increased blood pressure levels. Physiologically, the content of brain dopamine is clearly asymmetric and an alteration of this bilateral profile, as occurs in Parkinson's disease, may led to multiple alterations, including mood disorders such as depression [33]. The coexistence of various neuropeptides in dopaminergic neurons is also known, so that alterations in the content of dopamine will presumably affect the functions carried out by these neuropeptides. In fact, Banegas et al. [32] not only demonstrate an increase in blood pressure in animals with their left hemisphere dopamine-depleted, but also that in these animals (mainly injured on the left side) the enzymes that degrade some of such neuropeptides, such as cholecystokinin (CCK) and Ang II [34], as well as their correlation with their motor behavior were also modified [35].

**Figure 2.**
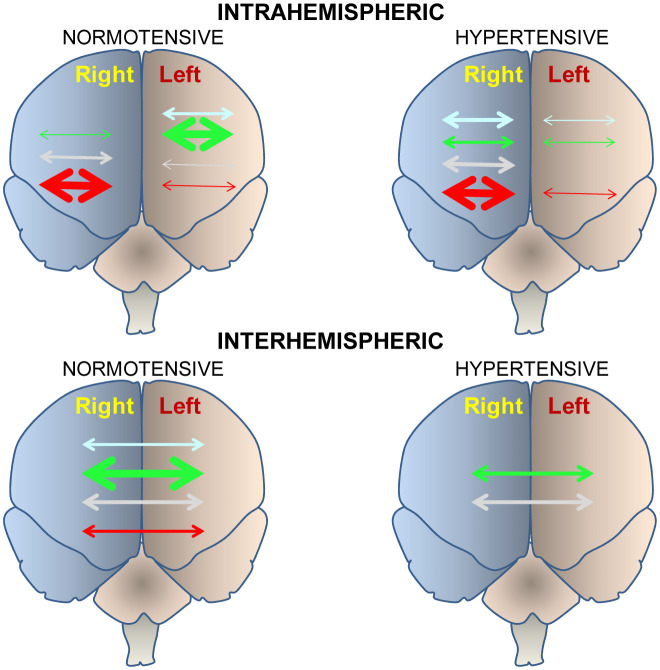
Intra and interhemispheric correlations.

If there is a relationship between central disorders and peripheral cardiovascular function and vice versa; and if this bidirectional interaction behaves asymmetrically, the analysis of the behavior of both hemispheres, particularly the frontal cortex, in the face of vasoactive hypertensive or hypotensive treatments could offer valuable data that would help us to understand this complex scenario. Thus, Prieto et al. [20] analyzed the behavior of various neuropeptide-degrading peptidases in the frontal cortex of animals treated with drugs that, through various mechanisms, are considered hypotensive, as it is the case of the inhibitor of the angiotensin converting enzyme captopril and the beta blocker propranolol or hypertensive drugs such as the nitric oxide synthase inhibitor, N (G)-nitro-L-arginine methyl ester (L-NAME), compared to the baseline conditions of normotensive or hypertensive animals without treatment. Their results were revealing since they showed a clear tendency to the right predominance in hypertensive compared to normotensive. This trend was also observed when analyzing the intrahemispheric or interhemispheric correlations between the different neuropeptidases that they studied (Figure 2). A greater number of intra-hemispheric correlations were observed in the left frontal cortex of normotensive animals treated with captopril. However, the increase in blood pressure after treatment with L-NAME gave rise to a marked predominance of intrahemispheric correlations on the right side. When we consider the interhemispheric correlations between the left and right frontal cortices, a high percentage was observed in normotensive animals treated with captopril. In contrast, in the hypertensive animals the interhemispheric correlations decreased drastically, which even disappeared in the groups with higher blood pressure levels, that is, in the untreated hypertensive animals and in those treated with L-NAME (Figure 2). In short, these results suggested that normotension or reduction in blood pressure after captopril treatment was related to high activity in the left frontal cortex, with a high left intrahemispheric correlation and a high interhemispheric correlation. However, hypertension, or an increase in blood pressure with L-NAME, was related to high activity in the right frontal cortex, with a high right intrahemispheric correlation and a decrease or absence of interhemispheric correlations. It is worth recalling now the work of Banegas et al. [32] in which the depletion of dopamine in the left hemisphere and not in the right one, led to a marked increase in blood pressure. The meaning of the prevalence or not of intrahemispheric or interhemispheric interactions is unclear [36] but its alteration with respect to normotensive baseline conditions may be an index of the onset or worsening of depression [37],[38].

Illustrative diagram showing the level of intra-hemispheric (top) or interhemispheric (bottom) interactions in normotensive (left) or hypertensive (right) subjects in untreated controls (blue) or treated with captopril (green), propranolol (gray) or L-NAME (red). The greater or lesser relative thickness of the arrows indicates respectively a greater or lesser number of significant correlations between neuropeptidases [20].

Following the brain-heart connection model, if the brain shows various bilateral patterns of behavior and correlation between neuropeptidases, in response to changes in blood pressure, this bilateral pattern could somehow be related to cardiovascular function and show some correlation between peripheral activity of the same enzymes at the plasma or cardiac level and at the brain level. The interest of the neuropeptidases analyzed in these studies lies in the metabolism of their potential endogenous substrates, which include central neuropeptides, neurohormones, local factors, and peripheral hormones (Table 1).

**Table 1.** Aminopeptidases and their actions. Enzymes whose activities were determined by Segarra et al. [18],[19] and by Prieto et al. [20]. Their abbreviations, EC Numbers and some of their most significant actions are indicated.

Name	EC Number	Action
Glutamyl Aminopeptidase (GluAP)	EC 3.4.11.7	Metabolizes Ang II to Ang III.
		Hydrolyzes CCK
Alanyl Aminopeptidase (AlaAP)	EC 3.4.11.2	Metabolizes Ang III to Ang IV.
		Hydrolyzes enkephalin
Cystinyl Aminopeptidase (CysAP)	EC 3.4.11.3	Identified as the AT4 receptor and the insulin regulated aminopeptidase (IRAP).
		Hydrolyzes Oxytocin and Vasopressin

**Figure 3.**
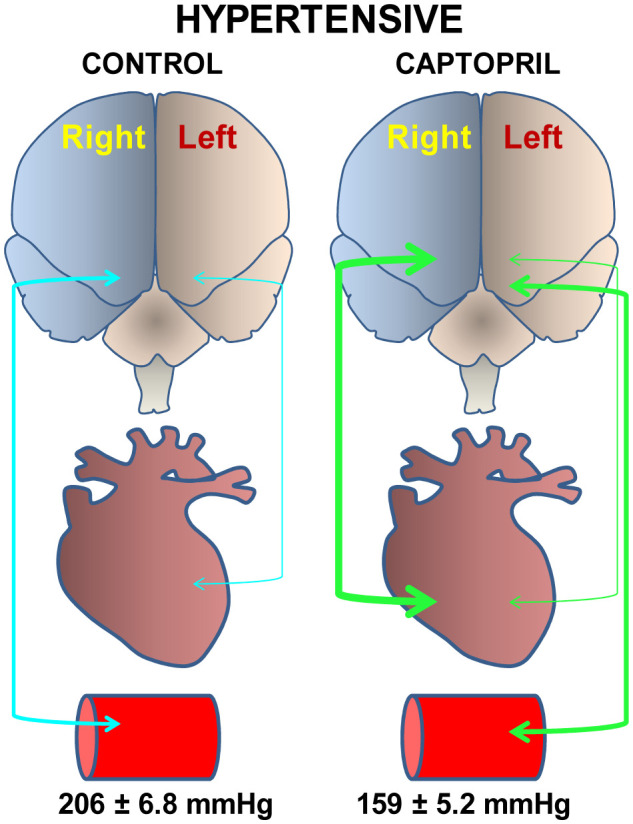
Bidirectional asymmetry in the brain-plasma-heart connection.

In untreated hypertensive animals with high systolic blood pressure (206 ± 6.8 mmHg), plasma aminopeptidase activities were significantly correlated with the right frontal cortex, but when hypertensive animals were treated with the angiotensin converting enzyme inhibitor captopril and decreased very significantly (159 ± 5.2 mmHg) their systolic blood pressure, the brain-plasma correlation moved to the left side [18]. In contrast, untreated hypertensive animals showed a significant correlation between heart and left frontal cortex, but in those treated with captopril there was a predominance of correlation between the heart and right frontal cortex [19] (Figure 3).

Correlation level between plasma or heart and the left or right frontal cortex in untreated hypertensive control animals (systolic blood pressure: 206 ± 6.8 mmHg) (blue arrows) and captopril-treated hypertensive animals (systolic blood pressure: 159 ± 5.2 mmHg) (green arrows). While in hypertensive control animals there was a correlation between plasma and right frontal cortex and between heart and left frontal cortex [18], captopril treatment inverted the correlation model, plasma correlated with the left side and the heart, mainly with the right [19]. The greater or lesser relative thickness of the arrows indicates respectively a greater or lesser number of significant correlations between neuropeptidases of one or another location.

This reciprocal and inverted behavior between plasma and heart with the left and right frontal cortex also suggests an asymmetry in the functioning of the autonomic nervous system, which has also been shown to use neuropeptides such as angiotensin that act as neurotransmitters in the autonomic innervation [39]. The results of Segarra et al. [18] connect with the observation that essentially the right hemisphere is the one that regulates cardiovascular function [40]. Inhibitors of the angiotensin converting enzyme are known to influence cardiac [41] and brain function, particularly by improving depression [29]. In addition, cardiac function influences brain function [42] and left or right brain lesions modify cardiac function [32]. Based on all of the above data, it seems evident that there is a reciprocal brain-cardiovascular functional connection and vice versa, with a neurochemical substrate, which is organized asymmetrically.

**Figure 4.**
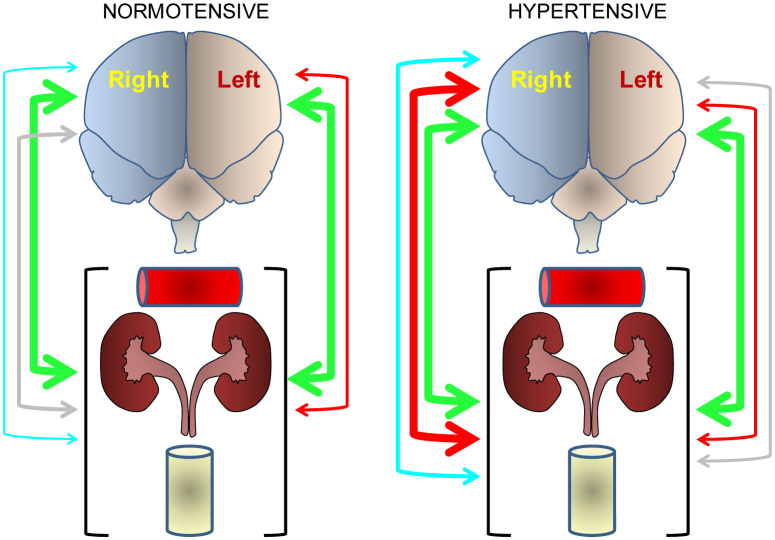
Asymmetry in the neurovisceral integration.

In basis to these results, the authors postulated that such asymmetrical brain-cardiovascular functional connection could be part of a global response of the organism that involves physiological parameters as well as metabolic factors in body fluids such as plasma or urine, framed within a global concept of asymmetrical neurovisceral integration [3]. To analyze this hypothesis, they studied the possible correlations between neuropeptidases of the left and right frontal cortices with physiological parameters such as water intake, diuresis or aqueous balance, metabolic factors such as lipids, nitric oxide and plasma glucose, as well as nitric oxide, proteins and creatinine in urine of normotensive and hypertensive animals treated or not with captopril, propranolol or L-NAME. The results obtained are outlined in Figure 4. Briefly, we can highlight that captopril treatment induces a bilateral connection between the left or right frontal cortex and the peripheral parameters as a whole, both in normotensive and hypertensive animals. In contrast, L-NAME treatment resulted in a high number of correlations with the right frontal cortex and only in hypertensive animals. The changes in the central-peripheral connection after the different treatments could be due to changes in the functional predominance of the left or right frontal cortex in normotensive or hypertensive animals. Changes in the bilateral behavior of the neurovisceral connection could be due to the predominance of nerve impulses that mainly involve the left or right frontal cortex to give rise to the secretion of neuropeptidases that regulate hormones that affect various peripheral parameters. These results have constituted the first observation of an asymmetric correlation between the left or right hemisphere with peripheral parameters, an asymmetric connection that depends on the animal's normotensive or hypertensive nature and the type of hypotensive or hypertensive treatment administered. The consequences of these observations are important since they suggest that, for example, in the case of a depressive state, it implies a certain bilateral cerebral pattern, accompanied by a specific and different functional/metabolic response to physiological basal conditions, and that such central bilateral pattern, along with the peripheral state, can be modified, tending towards or moving away from it, depending on the treatment that is administered to the subject.

Compendium of the results obtained by Segarra et al. [3] in which the correlations obtained between the left or right frontal cortex and the various physiological and metabolic parameters, considered as a whole, in normotensive and hypertensive animals are reflected in a simplified way. The color of the arrows indicates the treatment the animals underwent: blue (untreated controls), green (captopril-treated), gray (propranolol-treated), red (L-NAME-treated). The red horizontal cylinder represents plasma factors, the kidneys represent physiological functions, and the yellow vertical cylinder represents urinary parameters. The greater or lesser relative thickness of the arrow indicates respectively a greater or lesser number of significant correlations between central neuropeptidases and peripheral determinations.

## 4. Gut-brain connection

Within the neurovisceral integration model, there is no doubt that the gut-brain connection, which involves the autonomic nervous system and the endocrine system [2],[43], may have a relevant role in maintaining homeostasis: the physiological conditions of the individual. Various studies connect the type of diet, particularly containing saturated or unsaturated fatty acids, with the regulation of blood pressure, increasing or decreasing it respectively [44],[45]. Furthermore, there are results suggestive that this connection could also be organized asymmetrically. An alteration of this balance could be part of the pathogenesis of multiple disorders, including mood disorders such as depression. For example, a significant negative relationship has been observed between some gastrointestinal symptoms and left-handed individuals [46], however the relationship is positive between these left-handed subjects and inflammatory bowel diseases [47]. Interestingly, left-handed individuals are significantly more likely to have depressive symptoms than right-handed individuals [48] and an association has also been observed between young left-handed subjects and some altered autonomic cardiac function, such as an abnormal QRS-T angle. [49]. On the other hand, it should be noted that while a diet high in saturated fat induces depressive behavior [50], omega 3 fatty acids could have a positive effect on depression, in part through mechanisms that involve brain asymmetry. Thus, n3 polyunsaturated fatty acids (PUFA) could exert an antidepressant effect by modulating the reuptake and metabolism of monoamines as well as the fluidity of the membranes [51]. It is well established that the content of monoamines, particularly dopamine, is asymmetrically distributed in the brain and that a unilateral alteration of this distribution is part of the pathogenesis of Parkinson's disease in which not only motor alterations appear but also it is accompanied by depression and autonomic disturbances [33]. Certainly, the use of a polyunsaturate's enriched diet, compared to other diets, significantly modifies the content of fatty acids in the frontal cortex, especially increasing n3 PUFA [52]. Furthermore, it has been described that alterations in the n3 PUFA content in the diet are associated with alterations in the bilateral cerebral behavior possibly responsible for cognitive disorders [53]. A connection between the gut microbiota and the left hippocampus has also been hypothesized after a period of feeding with a diet enriched with sugar. Although a direct influence of sugar on the brain can be suspected [54], we cannot rule out the influence of a specific microbiota conditioned by a diet enriched with sugar [55].

## 5. Conclusions and perspectives

Within the concept of neurovisceral integration, the brain-heart connection is one of the most studied. From the initial concept postulated by Claude Bernard [16] to complex current analyses that try to relate cardiac function to brain function [42] as well as observations that demonstrate a neurochemical substrate for this connection and the significance of its asymmetrical behavior [3], the concept of “brain-heart connection” has become more complex but also, as a consequence, more interesting.

But what is the functional meaning of all of the above? We can speculate that the pattern of asymmetry in neurovisceral integration is also a dynamic concept, physiologically modifiable by endogenous and exogenous factors, and equally modifiable by pathological conditions. A certain dynamic bilateral profile under physiological conditions could intervene as part of the organism's homeostatic balance. Changes in this profile would lead to pathological outcomes. The organism, through various strategies, would tend to recover that lost balance. In the case of the relationship between the brain-heart connection and depression, there is a certain cerebral lateralization predominantly right in the depressive state. This lateralization is not only functional but also a neurochemical substrate underlies and a relationship has also been demonstrated between the increase in blood pressure and the development of depressive symptoms. Some antihypertensive drugs not only improve blood pressure levels through peripheral mechanisms, but also improve depressive symptoms, perhaps in part due to a tendency to recover the bilateral brain pattern. In addition, hypertensive drugs further increase blood pressure and reinforce the bilateral pattern of depression. On the other hand, certain nutrients induce a certain bilateral pattern that could counteract the one of depression. Therefore, pharmacological and/or dietary strategies that help to recover the baseline profile could constitute new tools in the treatment of depression.
